# Intron Evolution and Information processing in the DNA polymerase α gene in spirotrichous ciliates: A hypothesis for interconversion between DNA and RNA deletion

**DOI:** 10.1186/1745-6150-2-6

**Published:** 2007-02-01

**Authors:** Wei-Jen Chang, Victoria M Addis, Anya J Li, Elin Axelsson, David H Ardell, Laura F Landweber

**Affiliations:** 1 Department of Ecology and Evolutionary Biology, Princeton University, Princeton, NJ 08544, USA; 2 Linnaeus Centre for Bioinformatics, Uppsala University, Box 598, SE 751 24 Uppsala Sweden; 3 Department of Biology, Hamilton College, Clinton, NY 13323, USA

## Abstract

**Background:**

The somatic DNA molecules of spirotrichous ciliates are present as linear chromosomes containing mostly single-gene coding sequences with short 5' and 3' flanking regions. Only a few conserved motifs have been found in the flanking DNA. Motifs that may play roles in promoting and/or regulating transcription have not been consistently detected. Moreover, comparing subtelomeric regions of 1,356 end-sequenced somatic chromosomes failed to identify more putatively conserved motifs.

**Results:**

We sequenced and compared DNA and RNA versions of the DNA polymerase α (pol α) gene from nine diverged spirotrichous ciliates. We identified a G-C rich motif aaTACCGC(G/C/T) upstream from transcription start sites in all nine pol α orthologs. Furthermore, we consistently found likely polyadenylation signals, similar to the eukaryotic consensus AAUAAA, within 35 nt upstream of the polyadenylation sites. Numbers of introns differed among orthologs, suggesting independent gain or loss of some introns during the evolution of this gene. Finally, we discuss the occurrence of short direct repeats flanking some introns in the DNA pol α genes. These introns flanked by direct repeats resemble a class of DNA sequences called internal eliminated sequences (IES) that are deleted from ciliate chromosomes during development.

**Conclusion:**

Our results suggest that conserved motifs are present at both 5' and 3' untranscribed regions of the DNA pol α genes in nine spirotrichous ciliates. We also show that several independent gains and losses of introns in the DNA pol α genes have occurred in the spirotrichous ciliate lineage. Finally, our statistical results suggest that proven introns might also function in an IES removal pathway. This could strengthen a recent hypothesis that introns evolve into IESs, explaining the scarcity of introns in spirotrichs. Alternatively, the analysis suggests that ciliates might occasionally use intron splicing to correct, at the RNA level, failures in IES excision during developmental DNA elimination.

**Reviewers:**

This article was reviewed by Dr. Alexei Fedorov (referred by Dr. Manyuan Long), Dr. Martin A. Huynen and Dr. John M. Logsdon.

## Background

**Figure 1:**
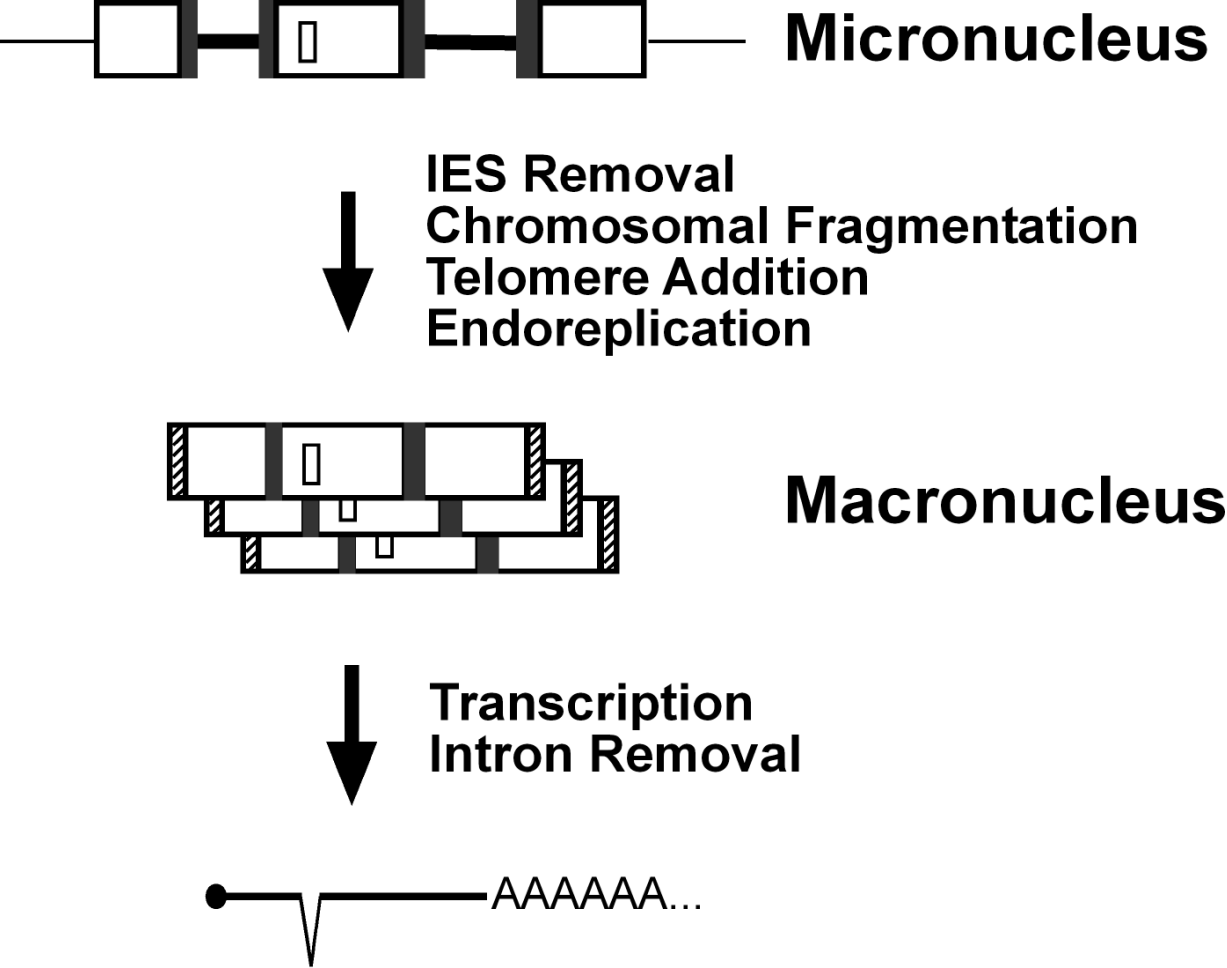
**Different layers of DNA and RNA processing in ciliates**. A schematic drawing of how germline (micronucleus) information is passed to soma (macronucleus). Genes, or macronuclear destined sequences (MDSs, large white open boxes), in micronuclear DNA are separated by internal eliminated sequences (IESs, thick black lines), flanked by pairs of direct repeats (grey boxes). Intergenic noncoding sequences are indicated by thin lines. After extensive DNA processing, IESs and intergenic noncoding sequences are deleted, MDSs are sewn together with one copy of each direct repeat retained. Telomeres (hatched boxes) are added to the ends and each macronuclear chromosome undergoes different levels of replication. mRNA transcribed from macronuclear chromosomes is capped (solid oval), polyadenylated, and a representative intron (small white box) is deleted.

Spirotrichous ciliates are intriguing because they carry out elaborate genomic DNA rearrangements when they develop their somatic nucleus, or macronucleus, from their hereditary nucleus, or micronucleus. In the hereditary nucleus, macronuclear destined sequences (MDSs) in the germline DNA are separated by noncoding, A-T rich, internal eliminated sequences (IESs), each bounded by a pair of direct repeats. Tens of thousands of IESs must be removed during macronuclear development, while the MDSs are "sewn together" leaving one copy of the pair of direct repeats at the "stitch" (Fig. 1, see also Table 1). After this event, the processed germline chromosomes undergo first heavy fragmentation, then end-capping with telomeres, and finally differential replication, to give rise to the macronuclear chromosomes or "nanochromosomes" (Fig. 1). In spirotrichous ciliates, the order and orientation of MDSs in the micronucleus can be completely different than in the macronucleus, in the remarkable illustration of "scrambled genes" [1].

**Table 1:** Comparisons of sequence removal via intron (RNA) versus IES (DNA) splicing.

**Repeat Pair Sequence^†^**	**Remainder After Removal as**		**Length Requirements to be Frame-Preserving**
		
	**Intron**	**IES**	
-X|**GT**ZAGY... ...XGTZ**AG**|Y-	XY	XGTZAGY	*Z*mod3 = 2^‡^
-XA|**GT**Y ... ...X**AG**|TY-	XATY	XAGTY	Impossible
-XAGZ**|GT**Y ... ... X**AG**|ZGTY-	XAGZZGTY	XAGZGTY	*Z*mod3 = 0

^† ^X, Y, and Z are arbitrary (potentially zero-length) sequences.

^‡ ^*Z*refers to the length in nucleotides of sequence *Z *and ''mod'' refers to the modulo operator.

The somatic macronucleus of spirotrichous ciliates contains millions of short macronuclear chromosomes ranging in size from ~400 to ~15,000 bp [2] and flanked by (C_4_A_4_)_n_C_4 _telomeric repeats [3,4]. Macronuclear chromosomes often contain a single gene, although some cases of multi-gene nanochromosomes have been reported [5-7]. A typical macronuclear chromosome contains the coding region of a gene plus short noncoding regions at the 5' and 3' ends.

Relatively little is known about the transcriptional structure and regulation of genes in spirotrichous ciliates, including their promoter sequences, transcriptional start sites, and signals that direct transcriptional regulation and post-transcriptional processing. A study of 66 putative protein-coding macronuclear molecules in spirotrichs revealed that the 5' and 3' flanking sequences are less than 200 bp and A-T rich (> 70%) [8]. A more recent analysis of 1,356 end-sequenced macronuclear chromosomes in *S. histriomuscorum *(formerly *Oxytricha trifallax*) also yielded similar conclusions [9], suggesting that signals for regulating transcription and/or translation, if any, are tightly packed in relatively limited spaces compared to other eukaryotes. However, the highly conserved eukaryotic promoter sequence TATA(A/T)A(A/T) [10] has not been consistently found in the 5' leader sequences, nor were conserved polyadenylation signals found in the 3' trailer sequences [2,8,11,12]. To date, only two *cis*-elements in macronuclear chromosomes have been experimentally confirmed: the heat shock responsive elements in *Sterkiella nova *(formerly *Oxytricha nova*)[13] and several *Euplotes *species [14] and the chromosomal breakage signal in *Stylonychia lemnae *[15,16]. Other putative *cis*-motifs deduced from multiple sequence comparisons include the putative chromosomal breakage signal 5'-HATTGAAaHH-3' (abbreviated using the IUPAC symbol H = not G) in *Euplotes crassus *[17] and a conserved AAGATA sequence present in 5' leader sequences of 24 completely sequenced actin-encoding macronuclear chromosomes in spirotrichs [18]. However, comparisons of more than 1,000 ends of macronuclear chromosomes revealed no conserved motif in the subtelomeric regions [9], with the exception of a purine-bias in the first 50 bp that Prescott and Dizick proposed might signal a chromosomal breakage during macronuclear development [19].

Spirotrichous ciliates generally contain few introns [2]. Known introns are present in both micronuclear and macronuclear DNA and, like IESs, are also A-T rich but bounded by canonical GT...AG splicing signals. An extended spirotrich-specific intron consensus of GTAAG...TAG has also been suggested [20,21]. Why introns are scarce in these taxa is unknown. Thus the bioinformatic discovery of introns in the DNA polymerase alpha gene [22] was unexpected.

In this study, we sequenced the genes and determined the mRNA ends of nine diverged orthologs encoding the large subunit of DNA polymerase alpha (pol α) in spirotrichous ciliates. We found a conserved motif aaTACCGCB (B = not A) located upstream from the transcription start site in each species. In addition, we also identified putative polyadenylation signals -10 to -15 bp upstream of the polyadenylation sites. We experimentally confirmed that introns in these genes were generally short, with the exception of two longer introns in euplotids. By comparing the locations of these introns within the coding region, our results suggest that independent gains/losses of introns have occurred within the spirotrichous ciliate lineage. Finally, we investigated the recurring and curious observation in our data of direct repeats overlapping or present near the exon-intron boundaries. Some of the larger of these repeats are similar in length to those that function in IES excision. We provide a statistical and bioinformatic analysis that shows that some of these direct repeats are more common than expected when compared to biologically constrained randomized genes. This result could be interpreted as strengthening a recent hypothesis (elaborated in [23]) that introns may evolve into internal eliminated sequences (IES). This would lead to their deletion from somatic chromosomes during macronuclear development, long before transcription. Such a novel pathway could explain the intron paucity observed in spirotrichous ciliates. An alternative interpretation is that some IESs might be selected to retain intron splicing signals, so that they can also be eliminated after transcription if IES excision fails [22,23]. We have observed that IES excision during macronuclear development is indeed error-prone [23] (Mollenbeck et al. unpublished), which makes such a mechanism for genome-wide intron/IES surveillance appealing.

## Results

**Figure 2:**
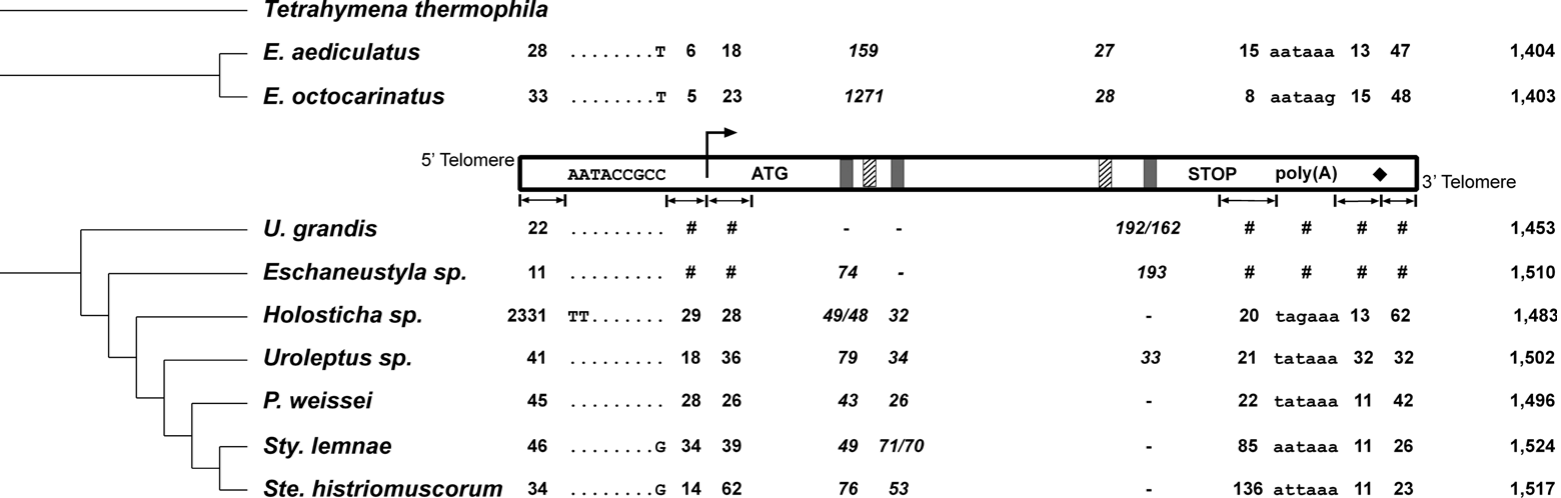
**Features of DNA polymerase α (pol α) genes from nine spirotrichous ciliates**. A schematic macronuclear DNA pol α gene is shown in the inset. This is flanked by telomeres. The inset also shows the consensus sequence of the 5' conserved motif (AATACCGCC), the transcription start site (right arrow), the putative translation start site (ATG), introns (3 found in seven stichotrichous ciliates as small grey boxes and 2 in *Euplotes spp*. as small hatched boxes), the putative translation termination codon (STOP), the putative polyadenylation signal sequence (poly(A)), and the mRNA polyadenylation site (solid black diamond). Although relative positions of these features are shown, they are not drawn to scale. *Italicized *numbers indicate intron lengths; allelic differences, when detected, are separated by "/". Numbers in the last column indicate the putative lengths (number of amino acids) of DNA pol α proteins from each species. Other numbers represent the distances from one motif to the next motif. For example, numbers in the first column represent distances from the 5' telomere to the 5' conserved motif. The symbol "#" indicates that data were not available due to the unavailability of RNA, while a dashed line ("-") indicates that the feature was not detected. Nucleotides in the 5' conserved motifs are shown as dots if they are identical to the consensus AATACCGCC. For each species, only nucleotides that differ from this consensus sequence are shown, as well as nucleotides that comprise the putative polyadenylation signal. A phylogenetic tree [18] is provided at the left of the figure for reference.

Features of the DNA polymerase α (pol α) gene of 9 ciliate orthologs are listed in Figure 2. The macronuclear sequences of this gene from two *Euplotes spp*. and *Eschaneustyla sp*. are newly reported in this study. We determined the mRNA ends from seven of the nine orthologs and re-sequenced the corresponding macronuclear regions in order to avoid possible discrepancies between RNA and DNA data due to strain differences or alleles within a species. RNA samples from *U. grandis *and *Eschaneustyla sp*. were not available; thus putative features were inferred from sequence comparison to other species.

### Conserved Motifs in 5' and 3' Flanking Regions of Nine Pol α Orthologs

**Figure 3:**
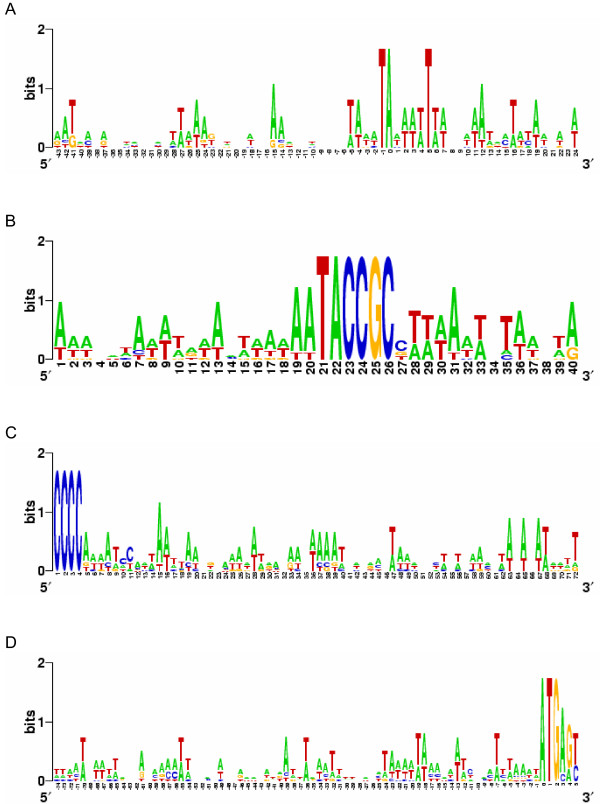
**Sequence logos of the 5' subtelomeric regions of the DNA pol α genes from nine spirotrichous ciliates**. Sequences were aligned at **A, **the transcription start site (position 0); **B, **the 5' conserved motif; **C, **the 5' telomere sequence; and **D, **the putative translation start site (ATG, position 0). Logos were calculated at [63].

All transcription start sites (TSS) were found on adenosine residues, preceded by a thymine base and followed by an A-T rich region (Fig. 3a). With the exception of the TSS in *Holosticha*, these sites are within the first 100 bp of subtelomeric DNA at the 5' end. In *Holosticha*, two other genes are present on the same macronuclear chromosome, upstream of the DNA pol α gene [6]. We searched 25 – 35 bp upstream of the TSS for "TATA box" sequences (TATA(A/T)A(A/T)) [10] and found no strong match, in agreement with conclusions of two previous studies [8,11]. However, we consistently found one copy of a 9 bp motif, aaTACCGCB, upstream of the TSS. This motif is also present in *U. grandis *and in *Eschaneustyla sp*. (Fig. 2 and 3b). The distance between this motif and the TSS ranges from 5 bp in *E. octocarinatus *to 34 bp in *S. lemnae*, while the distance between the end of the 5' telomere and the motif varies from 11 bp in *Eschaneustyla sp*. to 2,331 bp in *Holosticha sp*., which contains 2 additional genes in this region (Fig. 2; [6]). This motif was not present elsewhere on either strand of the macronuclear *pol *α sequences, and a search for this sequence in subtelomeric regions of 1,356 end-sequenced macronuclear chromosomes of *S. histriomuscorum *[9] also failed to identify more chromosomes carrying this sequence (Cavalcanti and Chang, unpublished results). We detected no other significant motif by aligning 5' non-coding leader sequences relative to the TSS (Fig. 3a), the 9 bp motif (Fig. 3b), the ends of 5' telomeric sequences (Fig. 3c), or the putative AUG start codons (Fig. 3d).

The lengths of the DNA pol α proteins, inferred from the nine orthologs studied, vary from 1,403 aa in *E*. *octocarinatus *to 1,524 aa in *S. lemnae *(Fig. 2). However, the seven conserved domains (I-VII) that characterize replicative DNA polymerases [24,25] and five domains (A-E) identified in eukaryotic alpha-polymerases [26] are all present in all nine genes described in this study. We note that a second, in-frame stop codon, a conserved feature in the 3' nontranslated region of 21 actin I genes in spirotrichs [18], was only present in the DNA pol α gene of *E*. *aediculatus*. Tandem stop codons appear to be under selection in yeast genomes to prevent read-through by a near-cognate tRNA [27].

The mRNA polyadenylation (poly(A)) sites of all seven DNA pol α genes were found at an adenine residue preceded by a thymine base, except in *E. octocarinatus *where a cytosine base proceeded the adenine (data not shown). Several previous studies have shown that the eukaryotic poly(A) addition signal AAUAAA [28] is not universally present within 50 bp upstream of the poly(A) sites [8,11,12,18]. Similarly, in our study, only two out of the seven DNA pol α mRNA sequences contain an AAUAAA sequence (Fig. 2). However, recent studies of human [29-32], mouse [32,33], and mammalian [29] expressed sequence tag databases revealed that fewer than 66% of mRNAs contain the classic AAUAAA. Single nucleotide variant AUUAAA and other potential sequences, including UAUAAA, AACAAA, UUUAAA, AAUAAG, AUUGAA, GUUAAA, ACUAAA and others, may serve as alternate poly(A) addition signals [33]. At least one of these putative signals is present in each of the DNA pol α mRNA sequences, within the last 50 bp upstream of the poly(A) site (Fig. 2). Another *cis*-element, a GU-rich or U-rich element downstream of poly(A) sites, has been shown to help guide the cleavage of pre-mRNA at the 3' end before poly(A) tail addition [34], and this element was also found in all seven mRNA sequences in this study (data not shown).

### Translation Initiation Sites and Introns

The translation initiation sites of spirotrich DNA pol α genes have been the subject of some controversy [35]. Ribosomal frameshifting, which has been demonstrated in *Euplotes *(for review, see [36]), or suppressed nonsense mutations have been proposed to repair the 5' end of the reading frame, which contains an in-frame stop codon near the previously assigned translation initiation sites in *S*. *lemnae *[35], *S. histriomuscorum*, and *S. nova *[37,38]. However, bioinformatic analyses suggested that two previously undetected 5' introns might provide a simple alternative explanation instead. The evidence for short introns came from analysis of patterns of synonymous and nonsynonymous polymorphism [22] and divergence among published macronuclear DNA sequence data (including preliminary data reported in this study) in all three possible reading frames, since the three exons occur in different reading frames. cDNA sequencing confirmed the presence of these introns. There are two phase zero introns flanking the region encoding conserved domain A in five stichotrichs: *Holosticha sp*., *Uroleptus sp*., *P*. *weissei*, *S*. *lemnae *and *S*. *histriomuscorum*. While intron length varies among these five species (Fig. 2), intron position is conserved in the protein alignment, with the first intron found at QYQ|V(D/E)E and the second at KLD|PNE (data not shown). With the exception of a hepta-nucleotide sequence TGCGGTA found in the first intron in *Holosticha*, *Uroleptus *and *Paraurostyla*, there is no significant sequence resemblance among "homologous" introns, despite the A-T richness of the intron sequences (65–88%). Through DNA sequence analysis, we infer the presence of the first intron in *Eschaneustyla sp*., but not the second intron. There is also no indication that either of these two introns is present in the early diverged stichotrich *U. grandis*.

We found only one intron in the 5' ends of DNA pol α orthologs in *E*. *aediculatus *and *E*. *octocarinatus*. The position of these introns in the protein alignment is 10 nt (or 3.33 aa) downstream from the position of the first 5' intron found in the other six stichotrichs (data not shown). Moreover, the intron is 159 bp in *E*. *aediculatus *and 1,271 bp long in *E*. *octocarinatus *(Fig. 2). To our knowledge, 1,271 bp is the longest intron identified in spirotrichous ciliates. We searched (blastn, blastx and tblastx) [39] Genbank and found no significant match to this long intron. It does not possess any detectable long inverted repeats that are often associated with transposable elements in *E*. *crassus *[40,41], nor does it contain ORFs longer than 70 aa. All introns in this study contain the canonical GT...AG splicing signal, but not all contain the putative extended splicing signal.

Upon removal of the intron sequences, we identified the putative start codons in each of the DNA pol α genes, corresponding to the conserved ATG noted in [35], and we also noted a conserved "MSD" at the very start of the translated protein. This motif is identical to the consensus sequence derived from other eukaryotic orthologs (KOG0970) [42], and we interpret this result as evidence for the true initiation site in the ciliate proteins. There is no indication of a programmed translational frameshift.

We identified one intron at the 3' end of the DNA pol α gene in *Uroleptus sp*.. This intron is 33 bp with no in-frame stop codon. We did not detect an intron at this position in the ancestral species *Holosticha*, nor in the later diverged *Paraurostyla*, *Stylonychia *or *Sterkiella*. However, the primary sequences of this gene in two other early diverged species, *U. grandis *and *Eschaneustyla sp*., suggest the presence of a longer 3' intron at the same position (Fig. 2). We also found one 3' intron in each of the two *Euplotes *orthologs. However, the latter are 81 nt upstream of the location of the 3' introns in three stichotrichs. It is therefore unlikely that the 3' introns in *Euplotes *and the three stichotrichs descended from a common ancestor, since this would require intron sliding.

### Intron Genealogy Comparisons

We compared the intron positions in the nine spirotrich orthologs and found evidence for independent intron gain or loss in this collection of taxa. For example, the second 5' intron is present in five later diverged stichotrichs, but absent from *Urostyla*, *Eschaneustyla*, and *Euplotes *(Fig. 2); the 3' intron in *Euplotes *is absent from seven stichotrichs; the 3' intron in *Urostyla *(Fig. 2), *Eschaneustyla*, and *Uroleptus *is absent from the other six species (Fig. 2). Notably, the DNA pol α gene contains three 5' introns in the related alveolate *Plasmodium falciparum *[43] and two in *Toxoplasma gondii *[44]. However, both of these apicomplexan DNA pol α proteins lack conserved domain A, precluding comparison of intron locations among all these species based on amino acid alignment (Addis, unpublished results). The DNA pol α genes from these two apicomplexan parasites each contain one 3' intron, but their locations are not conserved with each other, and both are present further upstream than the 3' introns in this study (Addis and Chang, unpublished results).

### Statistical Significance of Direct Repeats Flanking Some Introns

We noticed a tendency in our data for the presence of direct repeats bridging exon-intron boundaries. Short sequence repeats from four to eight nucleotides tend to overlap with or lie near intron donor and acceptor splice sites in six of the 14 introns we characterized (Table 2)[23]. The longer of these intron-flanking direct repeats resemble the sequence structure of internal eliminated sequences (IESs) that interrupt genes in the germline DNA and are excised during macronuclear development in ciliates. Conventional IESs are always bound by pairs of direct repeats, or pointer sequences, which are thought to guide proper assembly of macronuclear-destined segments [1]. Moreover, both introns and IESs are A-T rich. This resemblance raises the possibility that some introns flanked by direct repeats could be removed at the DNA level as IESs or *vice versa*, that some IESs could be removed at the RNA level as introns. Not all introns or species had intron-flanking repeats longer than 2 bp. However, for the purposes of statistical analysis we report in Table 2 the longest repeat that we could find in a 5 bp radius about intron-exon boundaries. In Table 2, we note that all of the repeat pairs of length five or greater (and one of length three) are biologically significant in the sense that they would preserve reading frame and not introduce stop codons if they were spliced out as IESs. Furthermore, the longer repeat pairs were generally unique in the macronuclear sequence and spanned only one macronuclear-destined segment (Table 2).

**Table 2:** Direct repeats found near intron/exon boundaries. The first six rows correspond to the first 5' intron, the next five rows to the second 5' intron, and the last three to the 3' intron.

Species	Intron	Intron length (bp)	5' repeat^†^	3' repeat	XL?^‡^	Location (MDS no.)	Freq.^§^
*Holosticha*	1	49	CAG < gta	cag < GTA	Yes	8	2
*Uroleptus*	1	79	TAG < gtt	tag < GTT	Yes	1	2
*Sterkiella*	1	76	TAG < gt	tag < GT	Yes	1	3
*Paraurostyla*	1	43	A < g	ag <	No	1–2	1936
*Stylonychia*	1	49	A < g	ag <	No	1	1871
*Eschaneustyla*	1	73	A < g	ag <	No	ND	1940
*Holosticha*	2	32	T < gta	tgtag <	No	8–9	15
*Uroleptus*	2	34	< gta	tag <	No	2	2178
*Paraurostyla*	2	26	TAGAT <	tag <	No	3	412
*Stylonychia*	2	71	AT <	atag <	No	2	2325
*Sterkiella*	2	53	AT <	atag <	No	2	2174
*Urostyla *I/II	1	192	< gtaaggcttatta	g< CTTATTA	Yes	41/41–42	2
*Uroleptus*	3	33	G < gtaa	g < GTAA	Yes	34	10
*Eschaneustyla*	2	193	AG < g	ag < G	Yes	ND	245

^† ^Symbolism: EXON < intron or intron < EXON. Repeats may be underlined for clarity.

^‡ ^XL?: translationally frame-preserving after excision as an IES

^§ ^Freq: frequency (%) of repeat word in entire MAC sequence

The question arises as to how likely such repeats would occur by chance, given the other constraints that influence these genes. The relevant constraints that we considered were protein function, codon usage bias, and intron splicing. For repeats of length four or greater, we generated random exonic sequences by permuting codons among synonymous sites, holding the translated amino acid sequence fixed. We combined these with randomized introns generated by permuting bases within each intron. We considered three different levels of constraint from intron splicing on donor and acceptor sites: no constraint; canonical eukaryotic, which held the first and last two bases of the introns fixed; and ciliate, which held the first five and the last three bases of the introns fixed. We refer to these as null models for calculating the probability of repeats. For smaller repeats we computed the expected frequencies according to these null models exactly (see methods).

All of the introns in our dataset contained canonical eukaryotic splice signals 5'-GT...AG-3' but they did not consistently contain the putative extended spirotrich ciliate splice signals 5'-GTAAG...TAG-3' referred to in [20,21]. Half of the introns in our dataset deviated from this extended consensus at one or more nucleotides and every site besides the first and last two bases was variable in at least one of the the 14 introns. Therefore, because we model only perfectly conserved signals, the most relevant null model we considered is the canonical eukaryotic one.

Our null models make the following assumptions: fixed amino acid sequences of the DNA pol α proteins in each species; fixed codon usage bias in the genes of each species with independent evolution of codons; and fixed base composition of each intron independently with independent evolution of bases and possible splicing constraints.

We then measured in the null models for each of the 14 introns how often we observed exact repeats of any word as long as or longer than the naturally observed repeats at the exact same positions bridging the exon/intron boundaries. The relevant results are shown in Table 3, where in each column, a False Discovery Rate (FDR) [45] is reported for the multiple comparisons across introns. Table 3 shows that repeats flanking the first and second introns in *Holosticha *and the first introns in *Uroleptus *and *Urostyla *are significantly rare in the eukaryotic splicing constraint null model with a false discovery rate of 10%. This implies that if the repeats flanking these four introns were to be investigated further, the expected risk is less than or equal to 10% that one of them is just a random occurrence as represented in this null model. In contrast, the eukaryotic splicing constraint null model could not be rejected for the generally shorter repeats flanking the other 10 introns with this FDR. Although the first three introns in Table 3 are homologous to each other, as are the last two introns, the Benjamini and Hochberg procedure is conservative when there is a positive dependency among tests [46].

**Table 3:** Estimated frequency of intron-flanking repeats as large or larger than observed in the natural data under species-specific random models of ciliate genes.

		Assumed intron splicing constraints		
		
Species	intron	None^†^	**Eukaryotic**^‡^	Ciliate^§^
*Holosticha*	1	42**	**2076+**	**4801~**
*Uroleptus*	1	60**	**2409+**	**5849~**
*Sterkiella*	1	148**	6848	14413
*Paraurostyla*	1^||^	*8129*	*72072*	*72072*
*Stylonychia*	1^||^	*7636*	*73737*	*737373*
*Eschaneustyla*	1^||^	*5928*	*81905*	*81905*
*Holosticha*	2	709	**408+**	**2583~**
*Uroleptus*	2^||^	*11191*	*17469*	*100000*
*Paraurostyla*	2^||^	*1415*	*26895*	*58273*
*Stylonychia*	2^||^	*12891*	*12891*	*27924*
*Sterkiella*	2^||^	*10739*	*10739*	*19570*
*Urostyla*	1	4**	**2+**	**0~**
*Uroleptus*	3	19**	6895	42397
*Eschaneustyla*	2^||^	314**	*81905*	*81905*

** FDR ≤ 0.01, + FDR ≤ 0.1, ~FDR ≤ 0.25: FDR (False Discovery Rate) controlled within each column by the method of Benjamini and Hochberg (1995), which was shown to control the FDR for positively dependent test statistics by Benjamini and Yekutieli (2001).

^† ^No intron splicing constraints: the entire intron was permuted.

^‡ ^Eukaryotic intron splicing constraints: the two bases at the 5' and 3' intron ends were fixed.

^§ ^Putative ciliate intron splicing constraints: the five 5'-most and three 3'-most bases of the introns were fixed.

^|| ^The values in italics were calculated exactly, multiplied by 10^5 ^and rounded; other values in upright face were calculated from permutation tests (N = 100,000).

As mentioned, we also calculated the frequencies of repeats in different null models of intron splicing constraints, one with no splicing constraints, and the other with extended splicing constraints. Even with extended "Ciliate" splicing constraints, the expected false discovery rate rejecting the null model for the same four introns is bounded by 25% (FDR ≤ 0.25). In fact, because of the evidence for this extended signal in our data and elsewhere [20,21], the true result is likely to be bracketed by those for the eukaryotic and ciliate extended models.

In all three null models we examined, the first *Uroleptus *intron was more likely to be flanked by repeats than the first *Holosticha *intron. The reason for this is that the *Holosticha *repeat contains a C, which is relatively rare in AT-rich ciliate genes, where *Uroleptus *presents a T. The fact that this substitution is mirrored in both repeat copies is consistent with their function in putative IES removal. Curiously, the TGTA repeats in the second *Holosticha *intron were significant in the eukaryotic model, despite a length of only four nucleotides. The reason for this is that the intron in question contains only one G, other than that of the intron splice acceptor. In fact, as discussed above and shown in Table 2, this repeat pair would not preserve frame if used in IES excision, nor does it lie within a single macronuclear destined segment. Therefore we do not consider it likely to function in IES removal.

We extended our results by looking for occurrences of the same repeat pair in the actual sequence data within 15 bp windows surrounding the intron/exon boundaries. We learned that these repeats occur only if they overlap the intron/exon boundaries in our randomized sequences, and almost always at exactly the same locations as in the actual sequences. As such, the simulated repeats were always frame-preserving when the natural repeats were frame-preserving. In searching for any repeat pair as long as or longer than those observed in the natural data, we found such repeat pairs of any sequence to to be enormously common. Only the 8-bp *Urostyla *3' intron repeats were still significant by these criteria (data not shown). However, all other repeats we studied, besides this one, fall tightly over the intron-exon boundaries, making the 15 bp window not applicable.

It is interesting to ask under what conditions repeat sequences in protein-coding regions can function dually either in IES removal or intron splicing, while preserving reading frame in both pathways. Considering only the generic eukaryotic splice signals GT and AG (since AT-AC introns have not been identified in ciliates), all possible variations are shown in Table 1, along with the conditions for such repeats to be frame-preserving. Pointer sequences containing the word AGT are never frame-preserving. The other two possibilities, words containing AG followed by GT, and words containing GT followed by AG, place different requirements on the length of the intervening sequence to be frame-preserving, as shown in Table 1.

## Discussion

The macronucleus of spirotrichous ciliates contains more than 20,000 short, linear DNA molecules flanked by C_4_A_4 _telomeric sequences (reviewed in [2]). These linear molecules form during macronuclear development by removing both *inter*- and *intra*genic spacer sequences from germline micronuclear DNA and then stitching gene fragments together. As a result, they contain mostly coding sequences with short flanking regions at both 5' and 3' ends [8,9]. Each macronuclear molecule is also present in multiple copies, ranging from 10^3 ^to 10^6 ^copies [2,47,48]. The advantage or disadvantage of this unorthodox genetic system is unclear. In addition, little is known about the molecular mechanisms that produce and regulate this system.

While the regulation of gene expression in spirotrichous ciliates is poorly understood, and *cis*-elements, such as promoter sequence(s), transcription factor binding sites, and poly(A) signal(s) have been difficult to find [8,11,12], most genes in spirotrichs have a consistent transcription start site (TSS) and poly(A) site [6,11]. Bender and colleagues replaced the α-telomere binding protein (α-TBP) gene in *E*. *crassus *with a gene for neomycin resistance and showed that this recombinant gene had the same TSS as the wide type α-TBP gene [49]. Furthermore, genes need to be precisely turned on and off during the ciliate life cycle, which suggests the existence of *cis*-elements to guide and regulate gene expression.

In this study, we compared gene sequences for DNA polymerase α from nine spirotrichous ciliates. We also sequenced RNA versions of seven of these orthologs and determined the mRNA 5' and 3' ends. We identified a motif of aaTACCGCB present 5 – 34 nt upstream from the TSS. This motif may serve one or more of the following functions: (i) promoter; (ii) copy number regulation, (iii) transcriptional regulator, (iv) chromosomal breakage signal. However, because this motif is absent from most other sequenced macronuclear chromosomes (Cavalcanti and Chang, unpublished results), it is unlikely to be a chromosomal breakage signal. Furthermore, the previously identified chromosomal breakage signals HATTGAAaHH in euplotids [17,50] and TGAA in *S. lemnae *[15,16] have no similarity to aaTACCGCB. While we have no evidence to support or reject any of the other three hypotheses, this motif is an excellent target for future functional studies of macronuclear gene regulation.

Several attempts to identify the poly(A) signal located at the 3' ends of macronuclear chromosomes have been reported [8,11,12]. Perfect matches to the eukaryotic poly(A) signal AAUAAA are not consistently found upstream of the poly(A) sites. However, as many as 40% of mammalian cDNA ends do not contain the AAUAAA signal [29-33] and several single-nucleotide variants of AAUAAA have been proposed as alternative poly(A) signals [33]. We searched for such sequences and found examples 10 – 15 bp upstream from poly(A) sites in the DNA pol α genes in six spirotrichs (vs. 32 bp upstream in *Uroleptus*) (Fig. 2). In addition, the candidate poly(A) motif (A/T)TAAAA derived from 27 *Euplotes *cDNA sequences [11] and the putative poly(A) motif TAAAC in *S. lemnae *[51] resemble the mammalian alternate signals. These observations suggest that a poly(A) signal is present at the 3' ends of most spirotrichous genes.

At 1,271 nt, the 5' intron in the DNA pol α gene in *E*. *octocarinatus *is approximately five times longer than the longest intron that has been previously reported in spirotrichous ciliates. In addition to the intron paucity in spirotrichs observed by Prescott [2], the spliceosomal introns identified in spirotrichs and in *Paramecium *[52] are generally shorter than 200 bp (for review, see [2]). While most research has focused on asking why introns in ciliates are short and how they are efficiently removed [53,54], the limitation on intron size raises the question of why long introns are rare in ciliates? A historical explanation could be that short introns were prevalent before ciliates diverged from other eukaryotes. Alternatively, there may be selective constraints that favor short introns in ciliates. We tested whether the long intron in *E*. *octocarinatus *derived from a transposon, a pathway that has been suggested by Rautmann et al. [55]. However, we detected no long inverted repeats [40,41] nor any putative ORFs that could partially encode a transposase [56]. Thus, the presence of this exceptionally long intron poses a small mystery.

We also noticed a quirky tendency of some introns in this study to be flanked by direct repeats, reminiscent of the pairs of pointer repeats that flank IESs and may facilitate their removal. In some cases the observed repeats flanking introns overlap intron donor and acceptor splice signals. Five repeats we found of length five or greater were biologically significant, in that they would preserve protein-coding information if they were to function in IES removal, and they also satisfy other criteria such as rarity in the sequence and linear contiguity in a single macronuclear-destined segment (Table 2). Furthermore, three pairs of the five biologically significant repeats were also statistically significant by the permutation criteria and tests we used to examine this question. The superficially "negative" result that arbitrary repeats are highly abundant in randomized ciliate genes raises the question of how the cell distinguishes those direct repeats that function as pointers in IES removal from those that occur randomly, and continually arise in evolution. The use of incorrect pointers in IES excision would likely lead to premature truncation of protein-coding sequences. Our results certainly suggest that further experimental pursuit of intron/IES dual function could be fruitful.

It is tempting to speculate that some ciliate introns may evolve into IESs by providing a nucleus for the evolution of extended repeats, which then lead to the elimination of the enclosed sequence during macronuclear development. This could help explain the observed intron paucity and some intron loss in spirotrichs during evolutionary time, as well as the shared locations of some introns and IESs [23,57]. Table 1 defines the sequence length requirements under which, within a protein-coding sequence, pointers that flank a nonscrambled IES might function equivalently in intron splicing without disrupting frame. This table may provide a useful way to study pointer sequences (in nonscrambled repeats) with possible or historical dual function. A further implication of our findings is that the proposed dual roles for such sequence repeats offers the possibility for surveillance of failures in IES removal. Examples of such a failure have been described in [22,58] and (Mollenbeck et al., unpublished). We speculate that the opportunity for surveillance and correction of errors could drive an advantage of dual function in either direction: If intron splicing were error-prone, then such circumstances could favor conversion of introns to IESs. Conversely, surveillance of faulty IES excision via sloppy intron removal would confer an advantage to the organism as well, by providing a second chance to restore reading frame.

## Conclusion

Our results suggest that conserved motifs, perhaps participating in gene regulation, are present in both 5' and 3' untranscribed regions of the DNA pol α genes. Our data also suggest that several independent gains and losses of introns in DNA pol α genes have occurred in the spirotrichous ciliate lineage. Finally, we found statistical and bioinformatic support for the hypothesis that direct repeats flanking introns might have an additional function in IES removal. This may favor the scenario in which introns evolve into IESs, which would explain the observed ciliate intron scarcity. Alternatively, some IESs might actually be spliced as introns, as a back-up in case they fail to be removed during macronuclear development. Further experimental pursuit of both hypotheses would be fruitful, noting that the two scenarios are not mutually exclusive and that both may contribute to the evolvability and robustness of information in ciliate genomes.

## Methods

### Cell Culture and DNA Isolation

*Euplotes aediculatus*, *Eschaneustyla sp*., *Holosticha sp*. [6], *Uroleptus sp*. [23], and *Paraurostyla weissei *[23] were isolated from lakes and soils in Princeton area and characterized by morphologies to the genus level. *Stylonychia lemnae *strain Y and *Euplotes octocarinatus *were generous gifts from Dr. Hans Lipps. *Sterkiella histriomuscorum *strain JRB310 was a generous gift from Dr. Glenn Herrick. DNA of *U. grandis *was a generous gift from Dr. David Prescott. Ciliate species were grown in conditions published elsewhere [6]. Macronuclei and micronuclei were separated before subsequent DNA extraction following established protocols [6].

### Macronuclear DNA Polymerase α Genes

Parts of the macronuclear DNA polymerase α (pol α) genes were first PCR amplified by using degenerate primer sets published elsewhere [35,37]. The PCR products were cloned, sequenced, and the newly obtained sequences were used to design specific primers and PCR amplify to 5' or 3' ends of genes following our established telomere suppression PCR protocol [6,59,60]. Genbank accession numbers for macronuclear DNA pol α genes are: *U. grandis *type I: AY008387; *U. grandis*type II: AY008386[23]; *Holosticha sp*.: AY293851[6]; *Uroleptus sp*.: AY293852; *P. weissei*: AY293806[23]; *E*. *aediculatus*: DQ060373; *E. octocarinatus*: DQ060372; *Eschaneustyla sp*.: DQ060374; *S*. *lemnae*: AF194338[35]; *S*. *histriomuscorum*: U59426[37]. Genbank accession numbers for other eukaryotic DNA pol α genes are: *Plasmodium falciparum *(PFD0590c); *Toxoplasm gondii *(AF093136).

### RNA Isolation and Rapid Amplification of cDNA Ends

Ciliate cells were fed with green algae one day prior to harvesting for RNA isolation. Cells were then concentrated by filtering through 10 μM sieve (Sefar American). RNA was extracted by using the Trizol LS reagent (Invitrogen) following the manufacturer's protocol. The quantity and purity of RNA were assessed by measuring UV absorbance at 260 nm/280 nm. 5'- and 3'- rapid amplification of cDNA ends (RACE) were carried out by using the FirstChoice RLM-RACE kit (Ambion) following manufacturers' protocols.

### Sequence Analyses

The alignments of DNA poly α genes were generated by using Accelrys (formerly GCG) software package. Sequence management under Windows OS was assisted by using the software Bioedit [61]. The 9 bp motif and direct repeats flanking introns were identified by inspection of the sequence alignment. We investigated the ability of each repeat pair to preserve reading frame after potential excision of their enclosed region as an IES. The alignment was trimmed and separated into exon and intron portions in Seaview [62]. DNA conserved motifs were calculated at [63].

### Permutations and Statistical Tests

Programs were written in Perl to generate permutations of the six sequences with observed intron-flanking repeats of length four or greater according to different constraints and to thereby test, by a randomization procedure, the statistical significance of the occurrence of repeats surrounding introns. Sequence manipulation was done in BioPerl [64]. Permutations were generated with the Fisher-Yates algorithm implemented in Algorithm::Numerical::Shuffle [65]. For each intron and exon of interest, sets of 10,000 or 100,000 random permutations were generated for statistical tests.

Codons were permuted within and among exons, preserving the amino acid sequence of the protein (using the UAR:Gln genetic code) and the codon usage of the gene. Bases were permuted within each intron either freely or fixing bases inside splice junctions, so as to preserve their lengths, base compositions, and potential splicing constraints.

For each repeat pair, permuted exons and introns were combined to generate simulated genes in which the occurrence of repeats was monitored.

For other species and introns with shorter repeats, the equivalent probabilities of repeats in our null models were computed exactly by hand from the probabilities of sampling words without replacement from multinomial distributions based on intron compositions and the codon frequencies of exons.

### Definition and Calculation of "Frame-Preservation" of Repeats Flanking Introns

Let φ_*Intron *_be the phase on removal of an intron and φ_*IES *_be the phase after IES excision from repeats that flank such an intron in such a way that this IES excision potentially disrupts the splicing of that intron (a disrupting pair). We say that the repeats are frame-preserving if φ_*Intron *_= φ_*IES*_. Let the 5' and 3' coordinates of the intron be (i, j), the 5' coordinates of a disrupting pair of repeats be A and C, and the 5' coordinates of a differently located disrupting pair of repeats be a and c. We then have φ_*Intron *_= (*j *- *i *+ 1)mod3, and φ_*IES *_= (*C *- *A *+ 1)mod3 or φ_*IES *_= (*c *- *a *+ 1)mod3. If the first pair of repeats are frame-preserving then (*j *- *i *+ 1)mod3 = (*C *- *A *+ 1)mod3, similarly for the second pair of repeats. The quality of frame-preservation is transitive; if both pairs of repeats are frame-preserving, then we know that (*c *- *a *+ 1)mod3 = (*C *- *A *+ 1)mod3. Since both (*c *- *a *+ 1) and (*C *- *A *+ 1) are congruent modulo 3, we know by definition that (*c *- *a *- *C *+ *A*)mod3 = 0. We used this last relation to test whether permuted repeats were frame preserving given that natural repeats are frame-preserving, without knowing the length or coordinates of the introns.

## Competing interests

The author(s) declare that they have no competing interests.

## Authors' contributions

WJC designed experiments, characterized DNA polymerase α sequences, analyzed genealogies of introns, and drafted the manuscript. VMA characterized mRNA and introns in stichotrichs. EA programmed and performed the statistical analysis. DHA inferred the presence of introns bioinformatically, designed the statistical analysis of repeats, derived the results in Table 1, proposed the hypothesis of intron surveillance of faulty IES excision, and helped draft the manuscript. AJL characterized mRNA and introns in the two *Euplotes spp*.. LFL designed experiments and helped to draft the manuscript. All authors read and approved the final manuscript.

## Reviewers' comments

### Reviewer's report 1

Alexei Fedorov, Department of Medicine, Medical University of Ohio (nominated by Manyuan Long)

This paper represents very interesting results and hypothesis, so I have no reservation in recommending it for publication. However, many places in this MS are not reader-friendly. I suggest a revision in the style of presentation (Minor revision prior to publication). In a nutshell, only a very small group of scientists have a good expertise in ciliates. Thus, a good brief introduction to the problem is desirable for a broader audience....

Author response: *We have now reordered and rewritten the background section for clarity and added a new figure *(Fig. 1) *to help a general reader understand the problem*.

On the other hand, when the authors start describing the introns on page 8, they present half a page of irrelevant discussion about suppression of non-sense mutations etc. I think that it is a nice piece for a lecture on this material. However, I was already overwhelmed by a number of specific details on the previous pages.

Author response: *We have shortened this section by simply presenting questions/hypotheses to be solved, followed by results*.

At the same time, since not many people know about ciliates' introns, it would be desirable to describe their general features. Do they have canonical GT.. ..AG termini? Is their splicing junction consensus similar to other species?

Author response: *So far all spirotrich introns are found to have canonical GT...AG termini, while extended signals for this group have also been proposed of GTAAG...TAG. We have included this information in the background and results sections*.

In addition, there is much room for improvement of the figures, in order to make them self-explanatory. Currently, a reader would need to spend some time in order to understand them.

Author response: *The new *figure 1* should be illustrative to a general audience and helpful toward understanding *figure 2.

Dr. Fedorov's minor comments are not shown.

### Reviewer's report 2

John M. Logsdon, Jr., Department of Biological Sciences, University of Iowa

Macronuclear copies of the DNA polymerase alpha gene from nine phylogenetically-diverse spirotrich ciliates have been analyzed here, including two new sequences for this study. The authors analyzed RNA transcripts from seven of these species in order to better understand cis-regulatory signals, and in doing so the authors have identified some spliceosomal introns in these genes. The manuscript details the analysis of conserved motifs, intron postions and statistical tests to evaluate the relationship between introns and internal eliminated sequences (IESs).

In a very straightforward manner, the results indicate some clear sequence motifs that are likely involved in transcription of these genes. This is a valuable contribution to the field since such signals are not well understood in these organisms. These ciliates are also somewhat depauperate of spliceosomal introns, making the authors' discovery of 2 or 3 introns per gene of some interest on its own. The presence of spliceosomal introns was verfied by cDNAs in most cases and reveals five separate sites that harbor introns. The two Euplotes species both have two introns (at shared positions) that are not shared in position with the introns in the other species. The three remaining introns are variably present among the other seven species: two are widely present (6/7 and 5/7), and one less widely (3/7). At face value, these data indicate that introns are being gained and/or lost in this lineage.

In considering a possible mechanistic relationship between spliceosomal introns and IESs, the authors evaluate some potentially shared signals between these processes. These statistical analyses suggest that introns flanked with direct repeats (as sometimes occurs with splicing signals) could be lost via IES excision (at the DNA level). The results are consistent with this interesting and novel hypothesis for intron loss, but it does not seem to explain many of their own observations. It is only the last (most 3') intron that requires an inference of loss in this dataset: this intron is present in Urostyla grandis, Eschaneustyla sp., and Uroleptus sp., and missing in all of the other taxa shown. Notably, this intron is missing in Holostricha sp., and in the P. weissei/Sty. lemnae/Ste. histrio. clade which, given the phylogeny, would indicate its separate loss in these two lineages. The other four introns can be parsimoniously explained by single gains with no losses.

Considering the putative intron losses from the Holostricha sp., and P. weissei/Sty. lemnae/Ste. histrio. clades, there is available data from some of these species on the micronuclear arrangement of this gene (PNAS 102: 15149). In these species, is there an IES in the same location as the "lost" intron? How about the other intron positions? In general, it might be useful to provide a "map" of the IESs relative to the intron positions.

Author response: *In the PNAS paper we did provide a map of IES locations (where pointers are) relative to intron positions. In some cases IESs coincide precisely with putative intron loss positions, e.g. an IES in P. weissei is present at the same position of the 3' intron in Uroleptus; numerous IESs surround the 5' intron regions in U. grandis*.

Also, the authors do not consider the converse idea: that some intron gain could be explained by the coincidence between splicing signals and IESs. That is, some unremoved IESs could function as spliceosomal introns.

Author response: *Although we had included this alternative interpretation in the conclusion, as it fits with our earlier work on the subject (Ardell et al. 2003), it was not expressed clearly enough in the version you reviewed. We agree with the reviewer about the importance of this alternative hypothesis and have now stated it more clearly throughout the paper*.

Although the authors argue that one of these introns (the first one) is shared between ciliates and animals (H.sapiens and C.elegans), the data presented are unconvincing. The alignment segment shown (Fig 3A) does not allow a clear assessment of homology of the residues involved. For example, the following alternative alignment in which a gap is shifted, seems possible:



INTRON POSITIONS/PHASE SHOWN ABOVE SEQUENCE)

The apparent gap in the C. elegans sequence casts doubt on the alignment (and homology assessment) of the intron-containing sites in animals. If the introns are not shared between animals, then there is very little reason to consider this position as "ancient" or even old. Thus, the apparently coincident position in the ciliate and human gene (which looks to be valid, given the alignment) would more easily be explained as separate gains into that site. In order to make a stronger case for the antiquity of this (or any other) intron, a much more phylogenetically broad survey of the available gene sequences and their introns will need to be performed and presented. If so, a more extensive alignment of the amino-acid sequences would be needed to assess the homology inferences at each of the intron-containing sites.

As for the other introns that are nearby known positions in orthologs from other species (as in Figure 3B), there is simply no good reason to consider these introns as homologous. There is very little (if any) support for the idea of "intron-sliding" as a mechanism to explain discordant positions. Instead, separate intron gain at nearby sites provides a better explanation for these data.

Author response: *We agree that our dataset may be too small to give a conclusive explanation about the intron gain/loss through representative eukaryotic species in this gene. We are deleting this particular comparison and will conduct a more thoroughly research with other genes in the future*.

### Reviewer's report 3

Martijn A. Huynen, Nijmegen Centre for Molecular Life Sciences, Radboud University Nijmegen Medical Centre, The Netherlands

The paper by Chang et al analyzes the DNA pol alpha genes in a number of spirotrichous ciliates. By comparing mRNA and DNA sequences the authors map the introns for these genes. Furthermore they examine their single gene chromosomes for the presence sequence motifs. Most interesting is the observation that they observe a preponderance of direct repeats spanning the exon-intron boundaries. Some of these resemble sequences that are eliminated during the development of the macronucleus, in which parts of the DNA are removed. This resemblance raises the possibility that the machinery that eliminates these DNA sequences operates also on introns or vice versa.

I have had some comments in an earlier version of this manuscript, but those have all been dealt with. Certainly in perspective of the enormous species diversity of ciliates and in perspective of their genome size (some contain more than 30.000 genes) there is relatively little known about these species with their peculiar genome organization. That their IES machinery is somehow related to their splicing machinery opens new possibilities for research. Having said that, the statistical support for the "non"-randomness of the direct repeats that the authors observe flanking the introns is rather weak. Furthermore not all the introns are flanked by repeats. The authors first identify the repeats and then test their significance. Nevertheless, the hypothesis is tantalizing and I do hope the authors will be able to find more support for it in future studies.

Author response: *We thank Dr. Huynen for his prompt response and his suggestion. As stated above, we would conduct a more extensive research with other genes*.
